# Dual Decline in Gait and Cognition Is Associated With Future Dementia: Evidence for a Phenotype

**DOI:** 10.1093/geroni/igaa057.846

**Published:** 2020-12-16

**Authors:** Manuel Montero Odasso, Mark Speechley, Richard Camicioli, Nellie Kamkar, Qu Tian, Luigi Ferrucci, Nick Bray, Frederico Pieruccini-Faria

**Affiliations:** 1 The University of Western Ontario, London, Ontario, Canada; 2 The University of Alberta, Edmonton, Alberta, Canada; 3 Parkwood Research Institute, London, Ontario, Canada; 4 National Institute on Aging, Bethesda, Maryland, United States; 5 University of Western Ontario, London, Ontario, Canada

## Abstract

BACKGROUND: The concurrent decline in gait speed and cognition are associated with future dementia. However, the clinical profile of those who present with dual-decline has not yet been described. We aimed to describe the phenotype and risk for incident dementia of individuals who present a dual-decline in comparison with non dual-decliners. METHODS: Prospective cohort of community-dwelling older adults free of dementia at baseline. We evaluated participants’ gait speed, cognition, medical status, functionality, incidence of adverse events, and dementia biannually over 7 years. Gait speed was assessed with a 6-meter electronic-walkway, and global cognition was assessed using the MoCA test. We compared characteristics between dual-decliners and non dual-decliners using t-test, Chi-square, and hierarchical regression models. We estimated incident dementia using Cox models. RESULTS: Among 144 participants (mean age 74.23 ± 6.72 years, 54% women), 17% progressed to dementia. Dual-decliners had a three-fold risk (HR: 3.12, 95%CI:1.23-7.93, p=0.017) of progression to dementia compared with non dual-decliners. Dual-decliners were significantly older with a higher prevalence of hypertension and dyslipidemia (p=0.002). Hierarchical regression models show that age and sex alone explained 3% of the variation in the dual-decliners group, while adding hypertension and dyslipidemia increased the explained variation to 8% and 10 %, respectively. The risk of becoming a dual-decliner was 4-fold if hypertension was present. CONCLUSION: Older adults with concurrent decline in gait speed and cognition represent a group at the highest risk of progression to dementia. These dual-decliners have a distinct phenotype with a higher prevalence of hypertension, a potentially treatable condition.

